# Morgagni Hernia in an Adolescent With Trisomy 21: An Uncommon Presentation in a Common Syndrome

**DOI:** 10.7759/cureus.90673

**Published:** 2025-08-21

**Authors:** Chaimae N'joumi, Hasnae Elhaddadi, Lamiae Maazouz, Imane Skiker, Abdeladim Babakhouya, Maria Rkain

**Affiliations:** 1 Department of Pediatrics, University Hospital Mohammed VI, Faculty of Medicine and Pharmacy, Mohammed First University, Oujda, MAR; 2 Department of Radiology, University Hospital Mohammed VI, Faculty of Medicine and Pharmacy, Mohammed First University, Oujda, MAR

**Keywords:** diaghragmatic hernia, down's syndrome, morgagni’s hernia, radiological diagnosis, trisomy 21

## Abstract

Morgagni hernia (MH) is a rare type of congenital diaphragmatic hernia (CDH), representing 1-5% of cases, and it often presents with nonspecific or incidental findings. We report a case of a 15-year-old female with trisomy 21 who presented with abdominal distension and intermittent fever. Imaging studies, including chest radiograph and CT scan, revealed a right-sided MH containing omental and colonic structures. The patient underwent successful surgical repair. This report emphasizes the importance of considering congenital diaphragmatic anomalies in patients with genetic syndromes who present with atypical symptoms, and highlights the crucial role of thorough radiologic evaluation for accurate diagnosis and management.

## Introduction

Congenital diaphragmatic hernias (CDH) are developmental defects of the diaphragm, with Morgagni hernia (MH) representing a rare anterior subtype, accounting for 1-5% of pediatric cases [1]. MH causes abdominal contents such as omentum, colon, stomach, or small intestine to herniate into the thoracic cavity [1]. The clinical presentation varies from asymptomatic to nonspecific gastrointestinal or respiratory symptoms, often leading to incidental diagnosis via imaging. Although rare, MH has been reported in patients with genetic syndromes like Down syndrome (trisomy 21), a population prone to multiple congenital anomalies, including cardiac and gastrointestinal malformations [2]. We present a case of a 15-year-old girl with trisomy 21 whose MH was unexpectedly identified during an assessment for abdominal pain and fever. This report emphasizes the importance of considering MH in syndromic patients with atypical abdominal symptoms.

## Case presentation

The patient was a 15-year-old female with trisomy 21 who presented with progressive abdominal distension, intermittent fever, and a mild non-productive cough. Her past medical history was notable for type 1 diabetes mellitus diagnosed at the age of nine, managed with conventional insulin therapy that had been inconsistently followed. There was no history of recurrent infections, gastrointestinal or respiratory symptoms, nor any known exposure to tuberculosis. Family history was non-contributory, but the patient reported consumption of unpasteurized dairy products. On clinical examination, she was hemodynamically stable, with a blood pressure of 105/65 mmHg, heart rate of 88 bpm, respiratory rate of 18 breaths/min, oxygen saturation of 98% on room air, and a temperature of 37.8 °C with a preserved general condition and exhibited phenotypic features consistent with Down syndrome. Physical findings included moderate generalized edema, most prominent in the face and abdomen, without peripheral limb involvement. The abdomen was distended with diffuse dullness to percussion, suggestive of ascites. Respiratory examination revealed decreased breath sounds at the right lung base, while cardiovascular evaluation was normal, with no audible murmurs. Small, non-tender cervical and inguinal lymphadenopathies were present.

**Figure 1:**
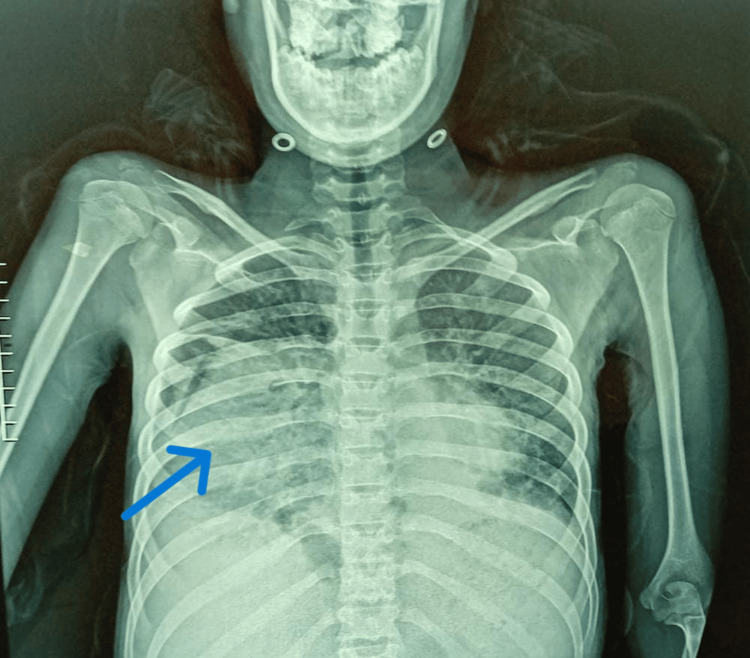
Frontal chest X-ray demonstrating a right lower lobe opacity traversed by air bronchograms (arrow)

**Figure 2:**
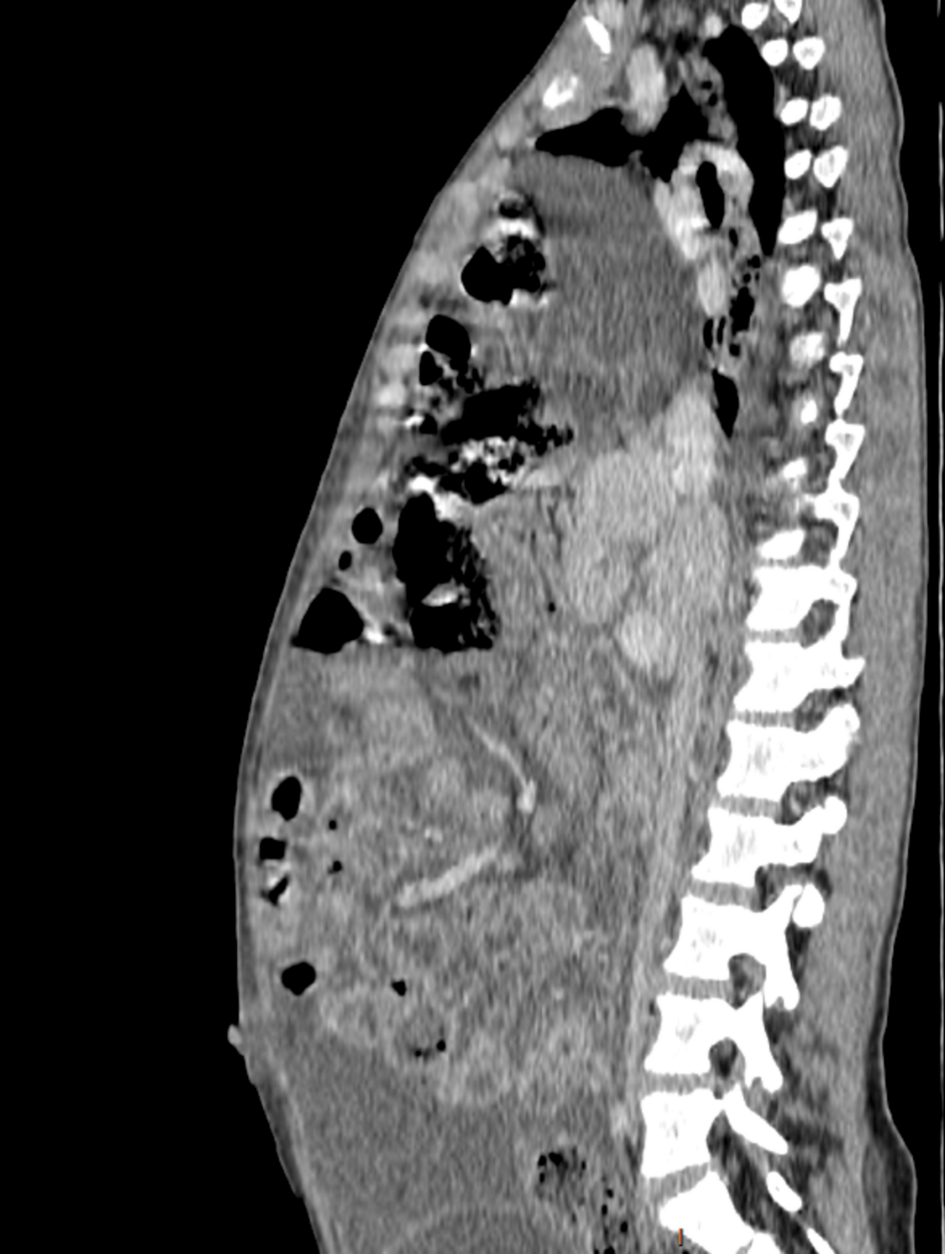
Sagittal contrast-enhanced CT of the chest and abdomen showing herniation of abdominal viscera into the thoracic cavity through the diaphragm CT: computed tomography

**Figure 3:**
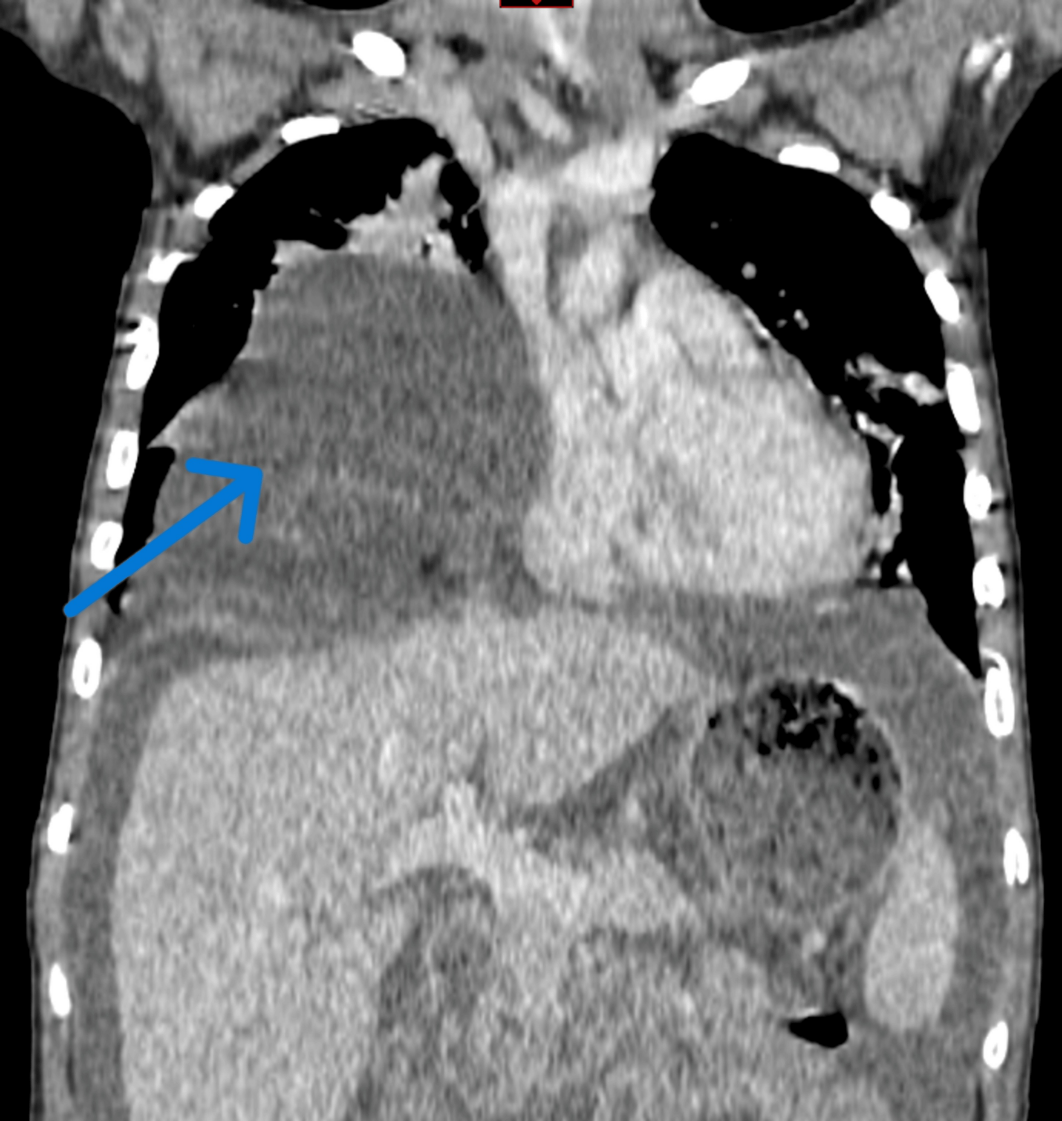
Coronal section of the thoracoabdominal CT scan showing the hernia (arrow) with slight displacement of left-sided structures, including the heart CT: computed tomography

Capillary blood glucose at admission was within normal limits. Laboratory evaluation revealed leukopenia with marked lymphopenia, elevated C-reactive protein, hypoalbuminemia, and otherwise preserved hepatic and renal function (Table 1). As part of the diagnostic workup for her abdominal and respiratory symptoms, a chest radiograph was obtained, which demonstrated a well-defined opacity in the lower third of the right lung field traversed by air bronchograms (Figure 1). This finding prompted a contrast-enhanced thoracoabdominal CT scan, which revealed a 75-mm anterior diaphragmatic defect through the foramen of Morgagni, consistent with a right-sided Morgagni hernia, with intrathoracic herniation of the greater omentum, ascending colon, and transverse colon. Additional findings included bilateral pleural effusions, supradiaphragmatic lymphadenopathy, and significant ascites (Figures 2, 3).

**Table 1:** Biological parameters on admission WBC: white blood cells; PNN: polynuclear neutrophils; CRP: C-reactive protein; AST: aspartate aminotransferase; ALT: alanine aminotransferase; GGT: gamma-glutamyl transferase; ALP: alkaline phosphatase

Parameter	Patient value	Normal range
WBC	3,670/µl	400,000 - 10,000,00/µl
Neutrophils (PNN)	2,930/µl	150,000 - 7 00,000/µl
Lymphocytes	420/µl	100,000 - 400,000/µl
CRP	65.40 mg/l	<5.00 mg/l
Albumin	26 g/l	35 - 50 g/l
AST	27 UI/L	5.00 - 34.00 UI/L
ALT	12 UI/L	0.00 - 55.00 UI/L
GGT	15 UI/L	7 - 32 U/L
ALP	109 UI/L	100 - 300 U/L
Urea	0.17 g/L	0.10 - 0.30 g/L
Creatinine	6.35 mg/l	5 - 11 mg/L

Diagnostic paracentesis yielded an exudative fluid with a lymphocytic predominance, raising the suspicion of abdominal tuberculosis. The patient subsequently underwent exploratory laparotomy for both diagnostic and therapeutic purposes. Intraoperatively, tissue biopsies were obtained, and the diaphragmatic defect was surgically repaired. Histopathological examination confirmed the diagnosis of abdominal tuberculosis. The patient was started on anti-tuberculous therapy, with a favorable clinical and radiological evolution. This case highlights the diagnostic challenges posed by atypical presentations in patients with underlying genetic syndromes and emphasizes the value of thorough clinical and imaging assessments. It further illustrates the incidental discovery of a Morgagni hernia in the context of an unrelated infectious etiology.

## Discussion

CDH is a rare developmental defect, with an incidence of one in 2,000 to one in 5,000 live births [3]. While most cases involve the posterolateral (Bochdalek) type, MH, located anteriorly at the junction of the septum transversum and thoracic wall, represents only 2-5% of CDH cases [4]. First described by Morgagni in 1761, this hernia typically contains abdominal contents such as the transverse colon, omentum, liver, and occasionally the stomach or small bowel [5]. In our patient, the hernia contained omental and colonic components. Morgagni hernias most often occur on the right side, as was observed in this case, likely due to the protective anatomical presence of the heart and pericardium on the left. Although rare, bilateral Morgagni hernias have been documented in the literature [1].

The etiology of MH remains largely idiopathic in over 70% of cases, though both genetic and environmental factors have been implicated. Single-gene mutations, de novo mutations, and teratogenic exposures-such as vitamin A deficiency or certain medications including allopurinol, mycophenolate mofetil, and lithium carbonate-have been reported in a minority of cases [6]. A strong association exists between MH and other congenital anomalies, particularly congenital heart disease and trisomy 21 (Down syndrome), with reported rates of associated anomalies ranging from 34 to 70% [2,3]. Down syndrome alone has been observed in 15-71% of MH cases across various series [4]. The occurrence of MH in identical twins with Down syndrome further supports a potential genetic predisposition [7]. The proposed mechanism includes defective dorsoventral migration of rhabdomyoblasts during embryogenesis, possibly influenced by altered cellular adhesion in trisomy 21, which may also explain the higher recurrence rates following surgical repair in this population [4].

MH often presents with nonspecific symptoms, thereby delaying diagnosis. In children, recurrent respiratory issues like chronic cough, dyspnea, and chest infections are the most common manifestations. Poor feeding, vomiting, and failure to thrive may also occur [8]. Gastrointestinal symptoms are less prominent but may still be present. Notably, up to 50% of cases are asymptomatic and discovered incidentally during imaging performed for unrelated reasons [9]. Adult presentations tend to include retrosternal pain, indigestion, bloating, or respiratory discomfort, often triggered by raised intra-abdominal pressure [4]. In rare cases, Morgagni hernias can present acutely with complications such as bowel obstruction, volvulus, or strangulation, requiring prompt surgical management [2].

The diagnosis of MH is primarily radiological, often triggered by incidental or nonspecific findings. Lateral chest X-rays may reveal retrosternal opacities or bowel loops in the right cardiophrenic angle, though hernias containing solid organs or with intermittent presentation can be missed [1]. CT remains the gold standard for confirming MH, offering nearly 100% diagnostic accuracy by clearly delineating the diaphragmatic defect and identifying its contents-typically omentum, bowel, or liver [4]. Additional modalities such as barium studies or MRI can support diagnosis in equivocal cases or when complications are suspected [4]. In prenatal settings, ultrasonography and fetal MRI can detect diaphragmatic hernias through signs such as mediastinal shift or herniated abdominal organs. Further investigations, like fetal echocardiography and karyotyping, are recommended to assess for associated anomalies, including congenital heart disease and chromosomal abnormalities like trisomy 21 [6].

Surgical repair is the definitive treatment, recommended even in asymptomatic cases to prevent life-threatening complications such as strangulation [3]. Both transthoracic and transabdominal approaches are used, with the latter generally preferred due to easier access and the ability to address associated anomalies [5]. Surgical strategy should be tailored to the patient’s condition, anatomy, and the surgeon’s expertise.

## Conclusions

Although uncommon, MH may present subtly or go undiagnosed until adolescence, particularly in individuals with trisomy 21, where symptoms can be atypical or masked by other health issues. This report highlights the need for careful clinical assessment and the use of appropriate imaging when patients present with nonspecific abdominal or respiratory complaints. Early diagnosis and prompt surgical repair are crucial to prevent complications and achieve positive outcomes.
